# A Concise Occupational Mental Health Screening Tool for South African Workplaces

**DOI:** 10.3389/fpsyg.2022.895137

**Published:** 2022-05-30

**Authors:** Charles H. Van Wijk, Jarred H. Martin, W. A. J. Meintjes

**Affiliations:** ^1^Institute for Maritime Medicine, Simon’s Town, South Africa; ^2^Division of Health Systems and Public Health, Department of Global Health, Faculty of Medicine and Health Sciences, Stellenbosch University, Stellenbosch, South Africa; ^3^Department of Psychology, Faculty of Humanities, University of Pretoria, Pretoria, South Africa

**Keywords:** psychological screening, occupational mental health, occupational health surveillance, South African workplace, workplace health

## Abstract

Mental health in the workplace is becoming of ever greater importance. General occupational health surveillance programmes are already in widespread use, with established referral systems for treatment and rehabilitation, and the same mechanisms could be expanded to include mental health screening and intervention. This study aimed to develop a concise composite mental health screening tool, based on analysis of existing data, for application in routine occupational health surveillance in South Africa. Data from workplace occupational health surveillance programs from 2,303 participants were analysed. Participants completed a number of questions/scaled items collated into a survey format, and partook in an interview with a psychologist. The data was analysed using frequency of positive self-reports, Chi square to calculate associations with outcomes, Receiver Operator Characteristic curve analysis to explore predictive ability, and binomial logistic regression to calculate the relative contribution of markers to outcomes. An exploratory factor analysis was further conducted on identified items. A general workplace model with 14 markers (and a maritime workplace model with 17 markers) were identified. The factor analysis suggested their organisation into five domains (similar for both models), namely neurocognitive health, common mental disorders, history of adaptation in occupational specific contexts, family-work interface, and stress overload. The study’s data-driven approach proposed a concise composite screener with less than 50 items, comprising five domains. This tool appears useful in identifying employees at risk for workplace injuries or poor mental health outcomes, and could be applied to related workplace settings in South Africa.

## Introduction

Mental health in the workplace is becoming of ever greater importance, from the perspective of both employees and employers. This article describes a process to develop a concise (i.e., comprehensive-but-brief) screen for mental health in the South African (SA) occupational context.

There is little comprehensive data available on mental health in the SA workplace. Recent local studies reported prevalence estimates for alcohol use disorder (7.2%), generalised anxiety disorder (5.0%), major depressive disorder (4.5%), and adult attention deficit/hyperactivity disorder (3.3%) in SA workplace samples, with little meaningful differences in prevalence across occupational categories (Van Wijk, 2020; Van Wijk et al., 2021b). Other SA studies estimated higher prevalences in high-risk occupational groups such as frontline medical personnel (Ward et al., 2006; Rossouw et al., 2013; Van Wijk et al., 2020) and police officials (Madu and Poodhun, 2006; South African Police Service, 2016). These groups are sometimes viewed as more vulnerable to adverse mental health outcomes, given their exposure to (potentially) traumatic workplace experiences, with high levels of post-traumatic stress disorder (PTSD) and problematic alcohol use reported. Recent reports from frontline medical staff during the COVID-19 pandemic highlighted the risk of poor mental health following extended exposure to traumatic experiences (Gold, 2020; Robertson et al., 2020; Zhang et al., 2020). In contrast to high-risk occupations, in general settings, work characteristics (such as decision latitude and effort-reward imbalance) appear to be more closely associated with mental disorders than occupational category (Stansfeld and Candy, 2006; Stansfeld et al., 2013; Chamoux et al., 2018). Broader socio-political contexts also matter, in that non-work experiences also impact on workers. For instance, South Africans face high prevalence of community level traumatic exposures and associated prevalence of PTSD, independent of occupational experience (Edwards, 2005; Kaminer and Eagle, 2010; Peltzer and Pengpid, 2019).

Poor mental health exacts a high toll on SA workplaces, through lost productivity and loss of earnings (Lund et al., 2013; Mall et al., 2015; Stander et al., 2016; Schoeman, 2017). This affects both organisational profitability and individual income, with the total annual cost to the SA economy calculated at more than R40-billion annually (Schoeman, 2017). Further, up to 50% of SA workplace accidents are thought to be related to substance abuse (McCann et al., 2011), and increased risk for workplace accidents and injuries have been reported where mental disorders are present (Kessler et al., 2009; Hilton and Whiteford, 2010; Palmer et al., 2014; Soares et al., 2018). Equally as important, individual workers suffer the personal distress associated with poor mental health in the workplace (Kessler, 2012; Lund et al., 2013).

Within the SA context, the Occupational Health and Safety Act (Act no 85 of 1993) places a responsibility on employers to monitor and manage workplace health and safety. In larger organisations this is often supported by tailored occupational health surveillance programmes. From a mental health perspective, workplace concerns are bi-directional (Van Wijk et al., 2021b): Firstly, occupational exposure may pose a risk for mental health injury (either by causally contributing to mental distress, such as PTSD, or by exacerbating existing mental health difficulties). Secondly, mental health difficulties affect workplace safety (e.g., by increasing the risk of accidents/injuries). As it is incumbent on employers to manage these bi-directional concerns, regular mental health screening may serve as a vehicle to support this (Harnois and Gabriel, 2002; Leão and Gomez, 2014; Neto et al., 2019).

Mental health screening could act as early warning of deteriorating mental health (and associated risks to an employee’s safety and wellbeing), as well as monitor the effects of workplace exposure on employee mental health (in order to intervene timeously to ameliorate the risk of workplace mental health injuries). As such it is mutually beneficial for both employer and employee, and could thus support the dual tasks of clinical management and workplace accident prevention. General occupational health surveillance programmes are in widespread use, with established referral systems for treatment and rehabilitation, and the same mechanisms could be expanded to include mental health screening and intervention.

However, screening for mental health in the workplace setting, using occupational health surveillance as vehicle, does pose a number of challenges.

(1) Lack of clarity regarding focus of screening. Mental health screening in the workplace is often plagued by poor definition of the constructs of interest (e.g., what is understood by ‘mental health’, but also many other). This is often linked to a poor understanding of the mental health spectrum (or ‘continuum’), which could range from severe mental illness to common mental disorders to mental distress to general emotional wellbeing. Poor understanding of this spectrum could result in a narrow screening focus that may only include severe mental illness, or general wellbeing, or other specific issues of interest, without accommodating the larger continuum. This may further be compounded by a poor definition of the purpose, or intended outcome, of screening, e.g., whether understood as improving safety, or productivity, or general worker/employee wellbeing. There may also be a poor conceptualisation of what information to include in screening initiatives. For example, should screening include historical biographical data (on the assumption that past behaviour is best predictor of future behaviour), or dispositional factors (such as personality), or history of past or current mental health symptoms, and so forth.
(2) The larger process within which screening is placed. Screening can only be used effectively and ethically if it occurs within a larger process with an established infrastructure to support referral and intervention. Workplace mental health promotion and prevention can be viewed as consisting of three components (Harnois and Gabriel, 2002), namely (mental) health education, screening for early detection and intervention, and action programs to address identified issues. As the purpose of screening is to allow for streaming of identified individuals for further investigation or management, it needs a robust referral system to manage identified concerns.
(3) Screening is resource intensive. Screening – particularly when coupled to a poor understanding of the constructs of interest – may be too broad (e.g., screen for too wide a range of constructs), or focus too narrowly, to be of productive use. To be economically viable, productive screening needs to be concise, that is, comprehensive-but-brief. This requires finding a balance between sensitivity and specificity concerns, whichever the tools used.
(4) Workplace culture and reporting reluctance. For open and honest disclosure to occur, respondents completing any screening tool should ideally accept the value of such an endeavour. For this to transpire, they need to understand the purpose of the screening process. This includes a clear understanding of the outcome, namely as supportive of mental health. This is in turn is closely associated with workplace culture, where the risk of stigmatisation needs to be actively countered. Further, extensive screening which becomes burdensome for respondents, risk decreasing acceptance of the process, making brief screening a more viable option.

Constructive mental health screening thus requires a tool that is comprehensive enough to encompass a meaningful spectrum of manifestations of poor mental health, but also brief enough to be practical and affordable. Such a tool needs to comprise items that are scientifically derived and clearly interpretable.

This paper describes the development of a tool for use in occupational mental health screening. It analysed data drawn from an existing database of workplace mental health screening initiatives, which included responses to a lengthy survey as well as to brief clinical screeners. Previous experience with the survey (which will be described under Methods) suggested that (a) the total survey is too long to be practical, and (b) that multiple domains appear to be involved, in that mental health was expressed as psychiatric disorders, or as poor emotional wellbeing, or in domestic discord or poor health behaviours, and thus potentially requiring a broader enquiry to elicit mental health difficulties.

The study therefore set the following aim, namely to develop a concise composite mental health screening tool, based on analysis of available data, for application in routine occupational health surveillance in SA.

The term *concise* is understood to refer to “comprehensive-but-brief,” while *composite* refers to the “multi-domain” nature of the screening tool. *Mental health* is defined “a state of mind characterised by emotional wellbeing, good behavioural adjustment, relative freedom from anxiety and disabling symptoms, and a capacity to establish constructive relationships and cope with the ordinary demands and stresses of life” (American Psychological Association, 2021). This refers to full spectrum or continuum, from general emotional wellbeing to severe mental illness, and include neurocognitive health. *Occupational health surveillance* refers to a “process that is regular (i.e., routinely conducted), with the aim of screening larger numbers of workers to identify cases requiring further attention” (e.g., intervention, referral, etc.).

## Materials and Methods

### Procedure

This study was a retrospective analysis of cross-sectional data. The data came from biennial, employer supported, workplace occupational health surveillance programs, where participants were invited to complete a survey (during the morning) and partake in an interview (during the afternoon). Respondents were allowed time of work to participate, but received no further incentive. Where appropriate, referral for further mental health support were arranged, fully sponsored by the employer. Ethics approval was obtained from the Health Research Ethics Committee of Stellenbosch University (#N20/07/078).

### Sample

A total of 2,303 participants provided data (not all participants completed all items, and the *n* for individual markers/items will be indicated in the Results section). Their mean age was 33.4 years (± 8.4, range 20–60), and 30.0% were women. Occupational field and gender distribution are presented in Table 1, as is home language distribution.

**TABLE 1:** Sample composition across gender and occupational field.

Occupational field	Women	Men	%
Navy sailors	111	254	15.8
Administrative/clerical	156	169	14.1
Security services	58	264	14.0
Qualified technicians (mechanical/electrical)	53	217	11.7
Marine officers	46	183	9.9
Survey recorders	72	83	6.7
Technical assistants (not formally qualified)	33	110	6.2
Hospitality/catering	51	79	5.6
Professional musicians	26	58	3.6
Unknown	85	195	12.2
**Total**	691	1612	100

**Language**	***n***	**%**	

English	438	19.0	
Afrikaans	402	17.5	
isiXhosa	278	12.1	
Setswana	278	12.1	
isiZulu	264	11.5	
Sesotho	244	10.6	
Sepedi	182	7.9	
Tshivenda	84	3.6	
SiSwati	49	2.1	
Tsonga	46	2.0	
Ndebele	32	1.4	
Other	6	0.3	
**Total**	2303	100	

All workers were in full-time employment at the time, with employer supported access to primary healthcare. The sample comprised semi- and skilled workers (e.g., in possession of either formal academic degrees/diplomas, or vocational training certificates), and who completed a minimum of 10 years of formal schooling (a requirement set to enable meaningful completion of the survey in English). The data was collected during 2019, prior to COVID-19.

A second sample was collected during 2021 (*n* = 672) using the same eligibility criteria. However, due to reduced screening during COVID-19, this sample was not representative of any workplace or industry, and only used here to confirm results of the main sample analyses. The mean age was 32.9 years (±8.3), 33.5% were women, and language and occupational distribution were similar to the main sample. They completed the same survey and interview, excluding the stress overload scale.

### Measures

All participants, as part of their general occupational health surveillance, completed a number of questions/scaled items collated into a survey format, and also partook in an interview with a psychologist later on the same day.

#### Survey

The survey consisted of four aspects: Firstly, it posed a range of questions regarding recent (e.g., past 2 years) history of psychological adjustment, including questions on previous mental health difficulties, workplace-specific adjustment, disciplinary issues at work, and interpersonal and related concerns. The history section consisted of items with YES/NO answers, and any YES answer would trigger further investigation.

Secondly, it enquired into neurocognitive health. Any YES answer on any neurocognitive item would trigger further investigation. Neurocognitive health data were only available for a subset of the sample (*n* = 574).

The three-item Simioni symptom questionnaire was originally developed as part of a first-tier screening for more severe HIV-Associated Neurocognitive Disorders (HAND) (Simioni et al., 2010; Southern African and Clinicians Society, 2013; Haddow et al., 2016). A score ≥1 previously showed fair sensitivity but poorer specificity for local samples (Joska et al., 2016). It was applied in the current study to identify cases requiring further investigation (α = 0.63 for this study).

The very brief two-item screen for adult ADHD (Zimmerman et al., 2017) was also developed as part of a first-tier workplace screening for more severe ADHD (Van Wijk and Firfirey, 2020), and a score ≥1 was used in the current study to identify cases for further investigation (α = 0.54 for this study).

Thirdly, current clinical symptoms were measured using four widely used brief screeners for common mental disorders. Any scale totals above the respective thresholds would trigger referral for further investigation.

The Patient Health Questionnaire-9 (PHQ-9) is a nine-item screening, diagnostic, and monitoring tool that measures the severity of depression (Kroenke et al., 2001; Gilbody et al., 2007). Local validation data supporting its use are available (Cholera et al., 2014; Bhana et al., 2015; Van Wijk et al., 2021a). A score ≥ 10 has been recommended as a positive screen for depression in low-and-middle-income contexts (Akena et al., 2012; Manea et al., 2012), and also considered optimal in international and local occupational health settings (Volker et al., 2016; Van Wijk et al., 2021a) and was used in the current study to identify cases of concern (α = 0.85 for this study).

The Generalised Anxiety Disorder questionnaire (GAD-7) is a seven-item screening, diagnostic, and monitoring tool that measures the severity of generalised anxiety (Spitzer et al., 2006; Löwe et al., 2008). Local validation data supporting its use are available (Bezuidenhout, 2018; Henn and Morgan, 2019; Van Wijk et al., 2021a). A score ≥ 10 was previously established as indicator for anxiety disorder in international and SA samples (Spitzer et al., 2006; Kroenke et al., 2007; Van Wijk et al., 2021a) and was used in the current study to identify cases of concern (α = 0.89 for this study).

The five-item primary care screen for post-traumatic stress disorder using DSM-5 criteria (PC-PTSD-5) was developed as a brief screen for PTSD in primary care settings using updated DSM-5 criteria (Bovin et al., 2021). It has demonstrated excellent diagnostic accuracy, with a score ≥ 3 offering optimal sensitivity and specificity in international and SA samples (Prins et al., 2016; Jung et al., 2018; Bovin et al., 2021; Van Wijk et al., 2021a) and was used in the current study to identify cases of concern (α = 0.71 for this study).

The four-item CAGE questionnaire was used to screen for problematic alcohol use (Dhalla and Kopec, 2007). Local validation data supporting its use are available (Claassen, 1999; Jung et al., 2018; Vissoci et al., 2018). A score ≥ 2 is generally applied (Dhalla and Kopec, 2007; Vissoci et al., 2018; Van Wijk et al., 2021a) and was used in the current study to identify cases of concern (α = 0.65 for this study).

Fourthly, current stress overload was measured with the 10-item Stress Overload Scale – Short Form (SOS-S) (Amirkhan, 2018; Wilson et al., 2018; Van Wijk, 2021), to indicate instances where a participant’s demands were experienced as overwhelming their available resources (α = 0.90 for this study). Previous SA research suggested that a score of >20 was associated with significant mental health difficulties (Van Wijk, 2021).

#### Psychological Interview

As part of the day-long comprehensive health assessment, each participant also partook in a semi-structured interview with a clinical psychologist. Interviews were concluded with referral or follow up arrangements, where appropriate. The clinical psychologists received comprehensive training and continuing supervision to support reliable interview outcomes.

The interview was used to determine “occupational mental health status,” which was used as the primary outcome variable for this study. Cases were allocated a binary code of FIT to continue working, or REFER if the interview elicited concerns about either safety at work and/or emotional wellbeing issues that needed to be managed through the occupational health referral system. This category was allocated after the interview assessed mental health against/within a very specific occupational context, which included the ability of an employee to safely do specific work in a specific context. This outcome category was operationalised as the “risk for adverse events (accidents/injuries) and/or poor mental health outcomes.”

### Data Analysis

All responses were entered onto an electronic spreadsheet. Biographical data were coded (e.g., gender, language), while nominal responses (YES/NO) were entered as is. The brief mental health screeners were summed and the total of each coded for meeting diagnostic threshold or not. As described above, a code was allocated to the outcome variable. The dataset was irreversibly anonymised prior to analysis. Analysis was conducted with statistical software, using SPSS-27.

There were a large number of variables involved, and the following analytical process was followed. Firstly, the frequency of positive self-reports (i.e., pick-up rate of items) were calculated as a percentage of the sample. A positive self-report was identified by YES responses for categorical questions/items, and score totals above diagnostic threshold for clinical screeners. Thereafter, associations with outcomes were analysed, using Chi square calculations.

This analysis was followed up by conducting a Receiver Operator Characteristic (ROC) curve analysis, using all individual and total-scale items that met the criterion of *p* < 0.25 on Chi square analysis (Bursac et al., 2008). This calculation was done to determine which markers meaningfully contribute to predicting outcome, for inclusion in future screening tools. The threshold for consideration of inclusion was set at AUC ≥ 0.6, the minimum threshold for acceptability (Yang and Berdine, 2017).

To calculate the relative contribution of markers to outcome, a binomial logistic regression (BLR) was conducted. This was to provide more accurate odds ratios when considering the relative/unique contributions of variables. However, the database comprised a long list of items, all binary in nature, and also not all participants completed all items (resulting in multiple missing items/scale totals). Variables were therefore selected for inclusion in the BLR following the guidelines of Choueiry (2021) and Heinze et al. (2018). After applying their criteria, it became clear that needed to separate a *general workplace* model from a *maritime workplace* model. Specifically, a number of variables were identified that referred to difficulties across various domains while “away at sea,” and applied to the ±26% of the sample that came from a maritime environment.

Thus, to determine variables for inclusion in a BLR for the general workplace model, the following, among others, was done:

1. Removed variables with low frequency (≤0.5%).
2. Removed variables that spoke to maritime specific context (i.e., 2 items).
3. Removed items with Chi square significance of *p* > 0.25 and AUC < 0.6.

After the four neurocognitive items were excluded (as only a subgroup of the total sample completed these items), the remaining 16 markers were entered into the BLR. The same procedure was followed to determine variables for inclusion in a BLR for the maritime workplace model, but retaining the additional two items with maritime reference (i.e., “difficulties while away at sea”). In this case 18 markers were entered into the BLR. An OR threshold of ≥ 1.5 for retention of markers were set as a practical arrangement.

In an attempt to further organise the identified markers, an Exploratory Factor Analysis (EFA) was conducted, with the scree test employed as the primary means to determine factors for retention.

## Results

Table 2 presents the results of markers that were associated with the outcome variable, including Chi square analyses, ROC analyses, and BLR. During the logistic regression, only variables with OR ≥ 1.5 were retained (Table 2), resulting in a general workplace model of 10 markers, and a maritime workplace model of 13 markers.

**TABLE 2:** Association and prediction of individual markers to at-risk indicator.

Items		Pick-up rate			X ^2^	ROC		BLR (general workplace model)			BLR (maritime workplace model)		
	*n*	Yes	No	%	(*p* < 0.001 for all)	AUC	95%CI	OR	Wald sign	95%CI	OR	Wald sign	95%CI
History of formal mood or anxiety disorder diagnosis	2,303	66	2,237	2.9	95.902	0.616	0.526 – 0.706	3.67	0.050	1.00 – 13.46	3.89	0.048	1.01 – 14.95
History of psychological/psychiatric treatment (past 2 years)	2,303	119	2,184	5.2	169.48	0.693	0.594 – 0.791	3.72	0.028	1.15 – 12.02	3.65	0.036	1.09 – 12.28
Suicidal thoughts or attempts in past 2 years?	2,303	30	2,273	1.3	166.61	0.604	0.514 – 0.694						
History of traumatic experiences at work (past year)?	2,303	57	2,246	2.5	134.79	0.628	0.537 – 0.0718	3.81	0.052	0.99 – 14.68	3.23	0.101	0.78 – 13.08
Concerns regarding use of alcohol	1,814	56	1,758	3.1	111.588	0.643	0.543 – 0.743						
History of treatment for substance abuse or dependency	2,303	11	2,292	0.5	32.050	0.522	0.430 – 0.614						
Previous significant personal adjustment difficulties at work (while away at sea)	2,303	67	2,236	2.9	51.487	0.585	0.497 – 0.674				4.91	0.024	1.23 – 19.61
Concerns regarding interpersonal relations in workgroup	2,303	106	2,197	4.6	98.048	0.647	0.557 – 0.737	2.53	0.079	0.90 – 7.10	1.86	0.257	0.64 – 5.40
Previous family problems (while away at sea)	2,303	22	2,281	1.0	26.152	0.535	0.450 – 0.620				1.50	0.678	0.41 – 3.65
Family crises that interfered with work (past 2 years)	2,303	108	2,193	4.7	123.617	0.666	0.576 – 0.757						
Disciplinary issues at work (past 2 years)	2,303	51	2,252	2.2	57.362	0.579	0.490 – 0.667				3.65	0.107	0.76 – 17.68
History of admission to hospital for psychological problems	572	12	560	2.1	28.505	0.607	0.429 – 0.786						
Frequent memory loss (past 3 months)	574	21	553	3.7	27.737	0.639	0.459 – 0.818						
Slower reasoning, planning, problem solving	574	37	537	6.4	49.556	0.742	0.573 – 0.912						
Difficulty sustaining attention	574	43	531	7.5	41.242	0.737	0.568 – 0.906						
Difficulty sitting still	571	48	523	8.4	35.674	0.732	0.564 – 0.901						
Domestic discord (past 3 months)	2,303	166	2,137	7.2	111.949	0.694	0.606 – 0.782	1.83	0.230	0.68 – 4.92	1.61	0.669	0.64 – 3.65
Major depression	2,303	84	2,219	3.6	518.308	0.792	0.705 – 0.879	6.17	0.003	1.86 – 20.44	10.55	0.000	2.90 – 38.46
Generalised anxiety	1,977	53	1,924	2.7	300.456	0.709	0.614 – 0.804	1.84	0.307	0.57 – 5.88	1.72	0.795	0.64 – 3.88
Post-traumatic stress disorder	2,303	50	2,253	2.2	452.494	0.719	0.630 – 0.809	5.37	0.005	1.66 – 14.44	5.85	0.004	1.75 – 19.55
Alcohol use disorder	2,303	157	2,146	6.8	96.926	0.676	0.587 – 0.765	4.53	0.002	1.73 – 11.86	3.65	0.013	1.32 – 10.08
Stress overload	1,811	419	1,392	23.1	102.023	0.833	0.774 – 0.891	3.80	0.042	1.05 – 13.75	3.39	0.079	0.87 – 13.19
Outcome: REFER	2,303	51	2,252	2.2									

*ROC, Receiver operator characteristic; AUC, Area under the curve; 95%CI, 95% Confidence interval; OR, odds ratio; BLR, binomial logistic regression.*

When conducting the EFA, Maximum Likelihood and Principal Axis Factors methods produced near identical outcomes, and the result of the maximum likelihood analysis are reported here. Five factors were retained – similar for both the general workplace (14 markers) and maritime (17 markers) models – which explained 60.26% of variance. A direct oblimin rotation provided the best solution. The results for the maritime model are presented in Table 3. Correlations between factors 1 to 4 ranged from 0.04 to 0.16, and correlations between factors 1–4 and factor 5 ranged from 0.10 to 0.42.

**TABLE 3:** Domains of mental health expression associated with psychological adjustment at work*.

Domain			1	2	3	4	5
1	Neurocognitive health (self-report symptoms)	Memory loss	**0.66**	0.17	0.23	0.09	0.15
		Slower reasoning	**0.30**	0.05	0.12	0.07	0.20
		Difficulty sustaining attention	**0.55**	0.02	0.06	0.06	0.03
		Difficulty sitting still	**0.67**	0.06	0.05	0.10	0.15
2	Common mental disorders (clinical history) (current symptoms)	History of mood or anxiety disorder	0.05	**0.86**	0.14	0.10	0.19
		History of psychological/psychiatric treatment	0.09	**0.66**	0.20	0.06	0.05
		History of traumatic experiences at work	0.21	**0.76**	0.02	0.12	0.16
		Clinical screeners PHQ-9	0.13	**0.83**	0.16	**0.30**	0.16
		GAD-7	0.07	**0.48**	0.06	0.05	0.08
		PC-PTSD-5	0.04	**0.82**	0.03	0.03	0.18
		CAGE	0.16	**0.46**	**0.30**	0.13	0.13
3	History of adaptation in occupational specific context	History of problematic interpersonal relations in workgroup	0.24	0.02	**0.52**	0.22	0.17
		History of poor adjustment at sea*	0.03	0.23	**0.65**	0.10	0.14
		History of poor discipline at work*	0.08	0.18	**0.94**	0.08	0.29
4	Family-work interface	Domestic discord	0.07	0.05	0.04	**0.48**	0.17
		Family problems affecting performance at sea*	0.10	0.16	0.12	**0.67**	0.27
5	Stress overload	SOS-S	0.12	0.20	0.12	0.09	**0.40**

PHQ-9, Patient Health Questionnaire-9; GAD-7, Generalized Anxiety Disorder scale-7; PC-PTSD-5, Primary Care screen for Post-traumatic Stress Disorder-DSM-5. *Maritime model only.

The second sample was analysed in the same manner, and confirmed the results of the main sample (not reported here).

## Discussion

This study aimed to develop a composite mental health screener, to ultimately identify (for referral and intervention where appropriate) employees at risk for adverse events (i.e., accidents/injuries) and/or poor mental health outcomes. The result, as described above, highlighted the role of markers of clinical mental health (i.e., psychiatric conditions) in identifying at-risk employees. The original long survey was administered in a health surveillance context and thus leaned toward clinical mental health, and this was reflected in the markers identified in this study. While it is recognised that mental health in the workplace is located within a much broader context of wellbeing (encompassing a wide spectrum), it stands to reason that more severe forms of mental health difficulties will be more predictive of poor outcomes, and thus would come to the fore in any analytic process.

The results further highlighted the value of a composite screener that covers multiple domains. In this regard, mental health screening tools may wish to incorporate the following domains:

(1) Neurocognitive health. This might be appropriate where environmental exposure at work (e.g., chemicals or gasses) may impact on brain health, or when neurocognitive deficits associated with chronic medical conditions may pose risks to the safe execution of workplace tasks. For example, SA has more than 8 million people living with HIV (Statistics South Africa, 2021), and the inclusion of neurocognitive markers could facilitate occupational safety through the early identification and subsequent management of HAND in the workplace context.

Neurocognitive health is also becoming particularly pertinent in the context of COVID-19. Firstly, the emergence of reports on post-COVID-19 neurocognitive difficulties (Daroische et al., 2021), even in mild or asymptomatic patients (Amalakanti et al., 2021; Graham et al., 2021), suggests a potential risk to workplace safety through increased risk of injuries and accidents. Secondly, particularly for frontline healthcare workers, COVID-19 poses a serious risk for occupational injury, with subsequent neurocognitive consequences. The inclusion of neurocognitive markers may be a critical tool in the early identification and ensuing management of COVID-19 Associated Neurocognitive Disorders (CAND) in the workplace.

It would be imperative that neurocognitive screening be appropriate and applicable to the specific work-requirements of an individual undergoing the screening.

(2) Common mental disorders. Past history together with current status – of both formally diagnosed disorders, as well as treatment for more general psychological problems – provided the strongest association with, and prediction of, increased risk for adverse events or poor mental health outcomes in the workplace. Appropriately validated brief screeners for common mental disorders – such as the scales in this study tapping mood, general anxiety, and alcohol use disorders – can be concise and effective tools for the identification of at-risk employees.

Further, comprehensive reports of increased incidence of mental health difficulties associated with the COVID-19 epidemic are emerging (Vincent et al., 2021; Xie et al., 2021), and brief scales such as these can be particularly useful in timeously identifying at-risk employees for further referral and intervention (Van Wijk, Under Review^1^).

(3) Adjustment in occupational specific contexts. The interview assessed mental health against and within specific occupational settings – in other words the ability of an employee to safely do specific work in a specific environment. Mental health at work cannot be separated from the specific work environment or larger organisational context (Harnois and Gabriel, 2002). In the current sample, reports of recent problematic interpersonal relations in workgroup were useful indicators of at-risk employees in the general sense.

Additionally, effective screening requires an awareness of occupation-specific contexts. In the current sample, specific contextual markers appeared useful in identifying at-risk employees in the maritime occupational environment (related to previous difficulties while at sea). Ships are often described as extreme, confined, and/or isolated environments (Suedfeld and Steel, 2000), and the additional demands of such an environment (including specific environmental and psychosocial stressors) (Jezewska et al., 2006; Oldenburg and Jensen, 2019; An et al., 2020) require close-knit teams and disciplined and well-adjusted individuals to successfully manage operations at sea. Markers of poor interpersonal relations, discipline, or adjustment in this context were significantly associated with risk. Where environmentally specific mental health screening tools are preferred – as is often the case in extreme, confined, and/or isolated environments – it may require adaptation to the specifics of individual workplace settings.

(4) Family-work interface. The complex intersection of family life affecting work and work effecting family life has been comprehensively described (Greenhaus and Foley, 2007; Appelbaum et al., 2009; Eby et al., 2010; Obrenovic et al., 2020). In the current sample, domestic discord was associated with an increased risk for accidents/injuries or poor mental health in the workplace. However, sensitivity is required when such markers are used to identify at-risk workers, as employees may wish to keep work and family life separate. The experience obtained when collecting the data for this study suggested that offers of referral to a social worker was generally accepted and associated with positive outcomes. However, not all employees may be open to such support, and an awareness is required to organisationally encourage the uptake of such services only when it affects workplace safety.
(5) Stress overload. In this sample stress overload was associated with an increased risk for accidents/injuries or poor mental health in the workplace, and as such was a useful marker for inclusion in a composite screener. However, this may not always be appropriate. Firstly, the term “stress” may be (mis)understood in different ways, while the condition of stress overload is often transient, and always subjectively interpreted. Secondly, the experience of stress may be associated with internal psychological mechanisms (e.g., poor coping skills) or external working conditions (e.g., high workload), and might require intervention on an individual level (e.g., to develop more effective coping strategies) or by an organisational specialist to evaluate and change, for instance, work-demands or resourcing. Thirdly, evaluation of stress overload may require more items than what a concise screener can allocate. It would be necessary to balance the added value of screening for stress overload with the conceptual and practical challenges that such a screener may bring.

A composite screen, as described above, that assesses markers across mental health domains, would be able to screen broadly enough to be clinically meaningful, yet be concise enough to be practically implemented. It is worth noting that it does not provide a total score to indicate need for referral or intervention, but rather provide markers across five domains of interest to guide further management. In this regard, within the neurocognitive domain, any YES answers would require further investigation. The same would apply to items in the workplace adjustment domain, where any YES would indicate the need for further investigation. The brief screeners for common mental disorders would be interpreted individually, according to the validated diagnostic guidelines for each.

This tool could serve two broad purposes within occupational health surveillance. Firstly, it could serve as a standard surveillance tool, administered at regular intervals (e.g., annually), in order to identify individuals who have developed mental health needs over time. Secondly, it could be used to monitor the effects of specific adverse and potentially stressful events at work, for instance by comparing post-event responses to baseline data. This tool may further be useful in the time period after the initial phases of the COVID-19 epidemic. The effects of the pandemic on occupational health issues have been comprehensively described (Giorgi et al., 2018; Lulli et al., 2021), and this composite tool could do duty both for regular screening and also to gauge the specific effects of COVID-19 on individuals and work-teams.

Indicators of concern should automatically trigger a referral process, where an identified individual is streamed to appropriate further investigation and intervention, which may include clinical intervention, or organisational intervention, and possibly a more frequent follow-up to monitor progress. The purpose would be to support an individual worker to optimally manage their mental health, both in the workplace and at home.

The importance of locating mental health screening within a broader approach of mental health promotion and prevention in the workplace cannot be overestimated. A robust multidisciplinary referral system is required, which may include clinical psychological or medical support for employees with mental disorders, or relationship counselling for employees with difficulties in their interpersonal relationships (whether organisational or domestic), or organisational intervention where employees report poor mental health due to job-stress (i.e., when the requirements of the job do not match the capabilities, resources or needs of the worker) (Harnois and Gabriel, 2002). As mentioned above, sensitivity is required – and hence the need for a broad approach to mental health promotion – to allow framing of referrals as supportive (e.g., to work safety or to enhance general wellbeing) as opposed to being viewed as interference into personal lives.

Screening tools are designed to identify at-risk employees. Additional mechanisms would be required to fully assess poor mental health and its association with workplace exposures within the context of identifying occupational injury, for purposes of determining both causation and compensation. The aetiology of mental disorders is located in complex intersections of biological, environmental, social, and psychological factors, and thus may occur for reasons not associated with workplace events. Further, only one DSM-5 disorder refers to environmental conditions as requirement for diagnosis (namely PTSD), supporting the principle that mental disorders are contingent on many factors, and not necessarily a response to the work environment. This is further confounded by the SA experience of multiple trauma exposure across life situations (also referred to as complex trauma), and determination of the contribution of workplace exposure to inform compensation lies outside the scope of occupational mental health screening tools.

### Limitations

This sample comprised semi- and skilled workers. It cannot be inferred that poor mental health in the workplace would be expressed in a similar manner in workers with different levels of training and skills. Further to this, SA is a multi-lingual society. Screening measures rely on respondents’ literacy with regard to the semantic descriptions of mental distress, and it is likely that employees without the English proficiency of the current sample might experience challenges in expressing their mental health experience in English. Future research would be invaluable to validate this and other concise composite screeners in different employment contexts and with different levels of skills and language proficiency. Future studies could also consider translated or adapted screeners for occupational contexts different from this study.

There is also concern that the internal reliability of the neurocognitive symptom scales is very low, particularly for the very brief ADHD screen, and its outcomes should be interpreted with caution.

## Conclusion

The need to develop concise mental health screening tools in resource-constrained contexts cannot be overstated. In South Africa, brief but valid and reliable mental health screeners have already proved important in both primary care and community settings in maximising the impact and (resource) efficiency of health surveillance-based interventions. In the SA occupational environment, where resources and mechanisms for proactive employee (mental) health surveillance and support are often non-existent, under-funded/capacitated, lack targetted specificity (or general applicability), or are resource intensive; these tools play an important role in minimising resource strain, such as employee/employer time, while still maintaining valid and reliable results. This makes concise-but-comprehensive mental health screening a far more attractive and practicable method of occupational health surveillance and support in SA workplaces.

This study aimed to develop a concise composite mental health screening tool, based on analysis of available data, for application in routine occupational health surveillance in SA. The study’s data-driven approach proposed a concise screener – with less than 50 items – that comprises five domains – thus establishing a composite tool. As such it combines both existing brief scales as well as markers from the lived experiences of SA workers in one tool. This screening approach appears useful in identifying employees at risk for workplace injuries or poor mental health outcomes, and could be applied to similar workplace settings in South Africa.

## Data Availability Statement

The raw data supporting the conclusions of this article will be made available by the authors, without undue reservation.

## Ethics Statement

The studies involving human participants were reviewed and approved by the Health Research Ethics Committee of Stellenbosch University. Written informed consent was obtained from all participants for their participation in this study.

## Author Contributions

CV conceptualised the study. CV, JM, and WM contributed to the analysis of data and were involved in the final review and editing of the manuscript. All authors contributed to the article and approved the submitted version.

## Conflict of Interest

The authors declare that the research was conducted in the absence of any commercial or financial relationships that could be construed as a potential conflict of interest.

## Publisher’s Note

All claims expressed in this article are solely those of the authors and do not necessarily represent those of their affiliated organizations, or those of the publisher, the editors and the reviewers. Any product that may be evaluated in this article, or claim that may be made by its manufacturer, is not guaranteed or endorsed by the publisher.

## Footnotes

1 Van Wijk, C. H. (Under Review). <jrn>Appraising Psychological Adaptation During COVID. (−)19 in South Africa: The Need for Multi-model Monitoring of Mental Health.
